# Hypertension in a patient with medullary sponge kidney: A case report

**DOI:** 10.1097/MD.0000000000024305

**Published:** 2021-01-22

**Authors:** Fengyuan Wu, Ying Zhang, Yunpeng Cheng, Yan Lu, Yinong Jiang, Wei Song

**Affiliations:** Department of Cardiology, First Affiliated Hospital of Dalian Medical University, Dalian, Liaoning, China.

**Keywords:** hypokalemia, medullary sponge kidney, secondary aldosteronism, secondary hypertension

## Abstract

**Rationale:**

Medullary sponge kidney (MSK) is a congenital renal disorder characterized by recurrent nephrolithiasis or nephrocalcinosis. Recently, it has been found that MSK can be also combined with other diseases, such as primary aldosteronism and Beckwith-Wiedemann, but whether it is associated with secondary hypertension remains unknown.

**Patient concerns:**

A 22-year-old hypertensive female presented to our hospital characterized by hypokalemia and hypertension.

**Diagnosis:**

The laboratory examination showed secondary aldosteronism. And the common causes for secondary aldosteronism include renal artery stenosis, glomerulonephritis, lupus nephropathy, and diabetic nephropathy, all of which were excluded except MSK.

**Interventions:**

She was treated with angiotensin-converting enzyme inhibitors.

**Outcomes:**

Her blood pressure, serum potassium, and plasma renin levels were reversed after treatment with angiotensin-converting enzyme inhibitors.

**Lessons:**

We presumed that MSK may be associated with secondary hypertension, and the mechanism may be the activation of the renin-angiotensin-aldosterone system.

## 1 Introduction

Secondary arterial hypertension accounts for about 5% to 10% of the general hypertensive population.^[1]^ Secondary hypertension should be screened if a hypertensive patient shows early onset of hypertension, resistant hypertension, severe hypertension and hypertensive emergencies.^[1]^ Hypokalemia in patients with hypertension accounts for about 15.8%, and, of these patients, secondary hypertension accounts for about 30.6%.^[2]^ The known etiologies of hypokalemia accompanying hypertension include primary aldosteronism, hypercortisolism, hyperthyroidism, and Liddle syndrome. Medullary sponge kidney (MSK) is a renal malformation known to be a benign disease. The estimated prevalence of MSK in the general population is between 1 in 5,000 to 1 in 20,000,^[3]^ while, in patients with renal stones, the incidence increases to 3%-5%.^[4]^ Whether MSK is an etiological factor for secondary hypertension has not been determined, although there are a few cases in which MSK is combined with hypertension. Here, we provide a young susceptive secondary hypertensive case without any etiological factors but MSK.

## 2 Case presentation

A 22-year-old female was suspected to have polycystic ovary syndrome (PCOS) due to experiencing menopause-like symptoms for two months. However, her menstrual cycle was recovered after treatment with ethinylestradiol and cyproterone acetate tablets (Dine-35) for 21 days. Two months later, during a resection of a fibroadenoma in the left breast, she was diagnosed with hypertension, and her blood pressure (BP) ranged between 150/110 to 180/110 mmHg. Meanwhile, her potassium concentration was 2.75 mmol/L. The patient had no symptoms - even when her BP was as high as 182/122 mmHg. There was no history of vomiting, diarrhea, or treatment with a diuretic. She was admitted for secondary hypertension screening due to hypertension combined with hypokalemia. Diltiazem hydrochloride sustained-release capsules and terazosin hydrochloride tablets were used to control the patient's BP. Physical examinations revealed that her temperature was 36.0 °C, her pulse rate was 70 bpm, her respiratory rate was 18 bpm, her BP was 171/104 mmHg, her body mass index was 18 kg/m^2^, her waistline was 70 cm, and the BP of her bilateral limbs was symmetrical. No clinical signs, such as a buffalo hump, moon face, or striae, were observed. There were no vascular bruits in the carotid and subclavian arteries, and there were no abnormal signs in the heart and lung. No renal vascular bruits was found upon abdominal examination. The pulsation of the radial artery and dorsal artery of the feet were regular and symmetrical.

Despite continuous oral potassium supplementation (3000 mg per day), her blood potassium was still low (3.33–3.53 mmol/L), and her 24-hour urine potassium was increased (72 mmol/24 h). Primary aldosteronism, hypercortisolism, hyperthyroidism, secondary aldosteronism (renal artery stenosis), and some of the genetic diseases, like Liddle syndrome, are the possible causes of hypertension combined with hypokalemia.^[5]^ However, hypercortisolism was excluded because her serum cortisol levels and the circadian rhythm of her cortisol secretion were normal. Hyperthyroidism was also excluded due to the normal thyroid function. Plasma aldosterone and renin concentrations were tested, which indicated that the patient's renin levels were increased (119.3 μIU/ml, reference range 4.4–46.1 μIU/ml), while her aldosterone levels were in the normal range (104 pg/ml, reference range 30–353 pg/ml). Additionally, the aldosterone-renin ratio (ARR) was 0.87 (normal range < 24). Primary aldosteronism and Liddle syndrome were excluded due to the increased renin activity.

**Figure 1:**
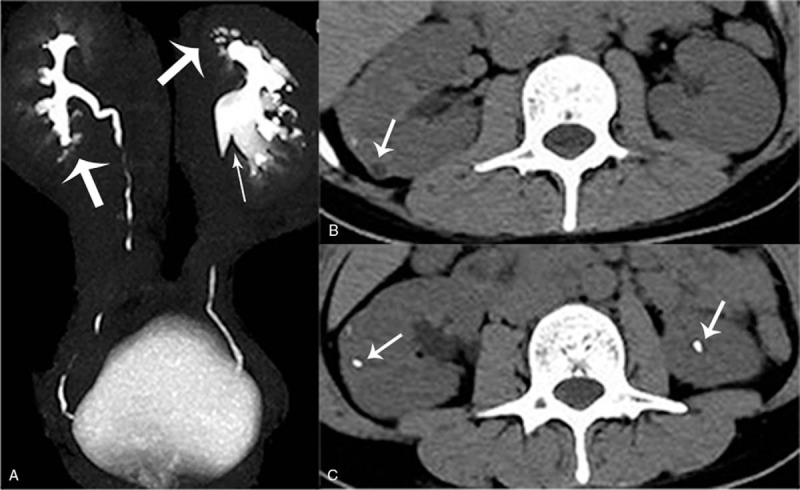
Computed tomography urography (CTU). (A) Dilated pelvis and calyces of right renal (thin arrow); characteristic imaging of dilated collecting tubules in bilateral kidneys with a “papillary brush” appearance (thick arrows); (B) Medullary cyst (arrow); (C) Enlarged right renal and multiple nephrolithiasis (arrows) in the bilateral renal parenchyma.

**Figure 2:**
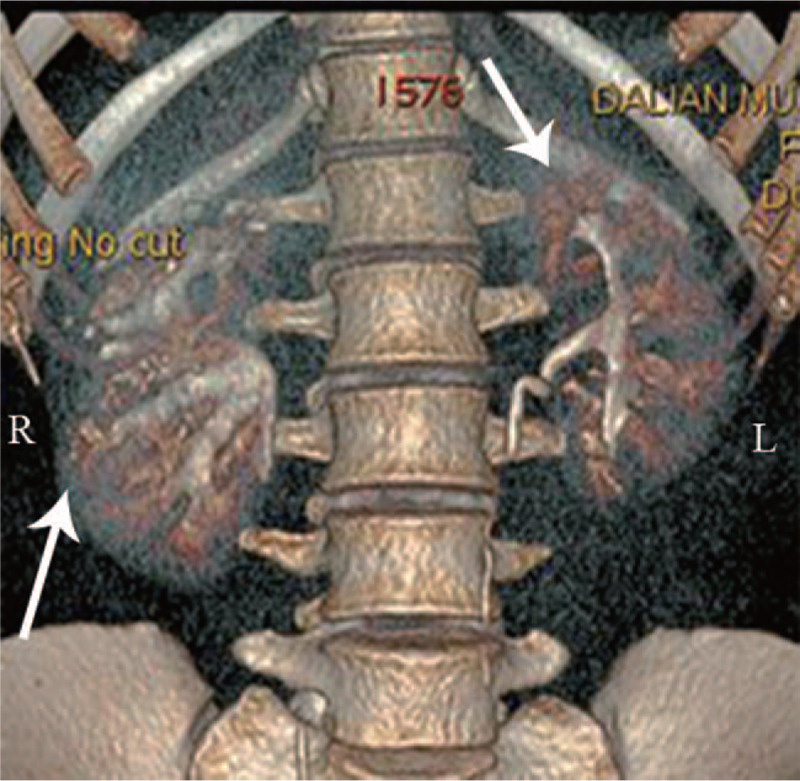
Reconstructed image of CTU with a “bouquet pattern” (arrows) in both kidneys, which was also the characteristic imaging appearance of the dilatation of the collecting tubules.

**Figure 3:**
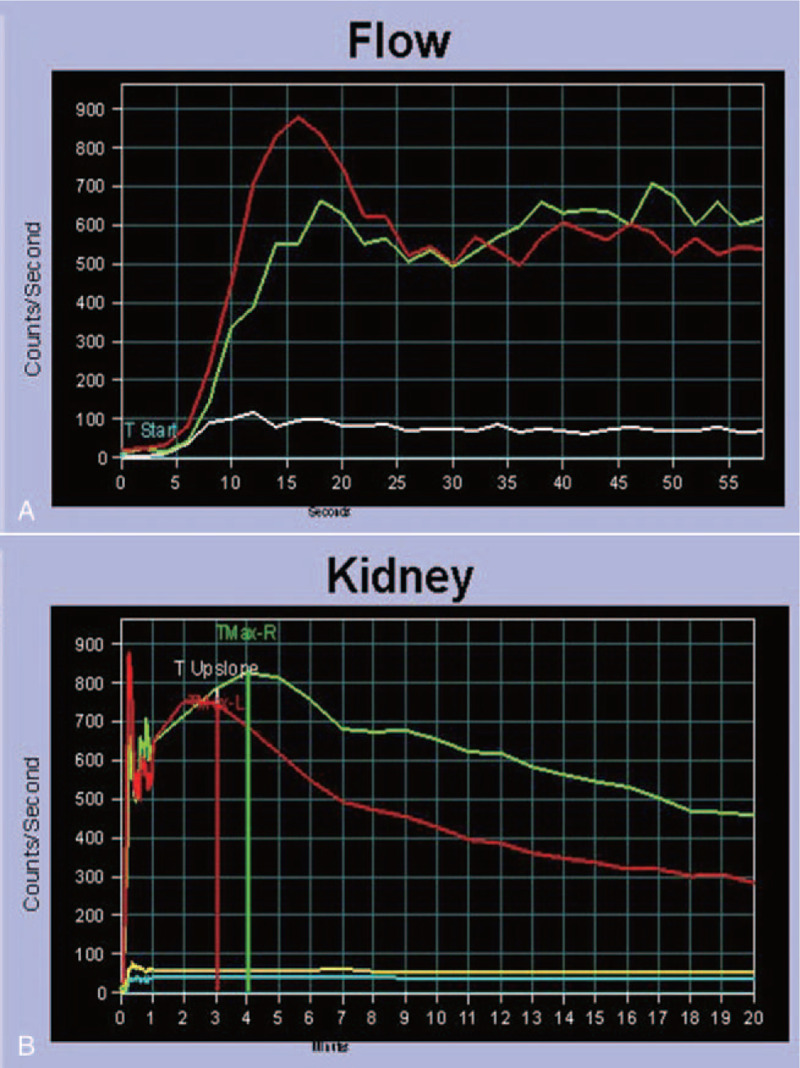
(A) The flow phase shows that the peak perfusion and perfusion velocity of the right renal (green curve) are lower than that of the left renal (red curve); (B) The elimination phase in the renographic curve shows that the time from the max counts to the 1/2 max counts of the right renal (green curve) is more than 20 min. Excretory function of the right renal is impaired.

The reason for the hyperreninemia needed to be investigated. One study indicated that hypokalemia may result in the depression of aldosterone secretion.^[6]^ When this was considered along with the patient's elevated plasma renin levels, it was deemed to be secondary aldosteronism. Renal parenchymal and vascular diseases are the common causes of secondary aldosteronism. While there was no renal artery stenosis confirmed by a computed tomography angiography (CTA) of the bilateral renal arteries, urinalysis indicated two to four red blood cells and three to five white blood cells per high-power field. The urinary albumin creatinine ratio was 238.73 μg/mg, and the urinary protein volume was 415 mg/24 h. The suspected causes of renal parenchymal diseases, such as glomerulonephritis, diabetic nephropathy, and lupus nephropathy, were suspected. Serum complement, serum anti-streptolysin O (ASO), plasma glucose, anti-dsDNA antibody, and anti-SM antibody levels were tested, yet all were either normal or negative. However, an abdominal ultrasound revealed multiple renal stones in the bilateral kidneys, right hydronephrosis, and an enlarged right kidney. Computed tomography urography (CTU) was performed in order to determine the reason for the hydronephrosis, and it revealed a dilated collecting system in the right kedney, a “papillary brush” appearance in the bilateral kidneys (Fig. 1A), medullary cysts (Fig. 1B), and multiple calcifications in both kidneys (Fig. 1C). The reconstructed image of the CTU specifically revealed a “bouquet of flowers” appearance (Fig. 2). Moreover, there was no urinary tract obstruction at the ureteropelvic junction, and the CTU demonstrated the typical manifestations of MSK. In order to evaluate the split renal function, 99Tc-DTPA renal dynamic imaging was performed. The flow phase showed that the perfusion volume and velocity of the right kidney was lower than that of the left kidney (Fig. 3A). The renographic curve revealed that the excretory function of the right kidney was impaired (Fig. 3B).

The patient was finally diagnosed with secondary hypertension related to MSK after other etiological factors were ruled out. Benazepril hydrochloride at a dose of 10 mg daily was prescribed for antihypertensive therapy. The patient's serum potassium was increased to 4.0 mmol/L without a potassium supplement, and her blood pressure was controlled to around 120/80 mmHg after a month of treatment. Plasma renin and aldosterone concentrations were restored to normal levels after three months.

## 3 Discussion and conclusions

The patient was suspected to be secondary hypertension due to spontaneous hypokalemia and the early onset of hypertension without a family history of hypertension. Other causes of hypertension with hypokalemia, such as primary aldosteronism, hypercortisolism, hyperthyroidism, and Liddle syndrome, were ruled out based on the increased renin activity, normal cortisol levels, and thyroid function. Secondary aldosteronism is also a common etiological cause of hypertension with hypokalemia, however, it can be masked by hypokalemia. Secondary aldosteronism is usually associated with renal ischemia caused by renal parenchymal and vascular diseases. However, the CTA of the bilateral renal arteries showed no renal artery stenosis. The patient had no history of infection, and her serum complement and serum ASO was normal. Glomerulonephritis was ruled out, and diabetic nephropathy and lupus nephropathy were also excluded according to the patient's history. Laboratory examinations showed microscopic hematuria and microalbuminuria. The microscopic hematuria was considered to be associated with the renal stones, and the microalbuminuria were perhaps related to the organ damage caused by the hypertension.

MSK is usually characterized by the tubular dilation of the renal collecting ducts and the cystic dilation of the medullary pyramids.^[7]^ CTU is recommended for the diagnosis of MSK, although the traditional method is intravenous pyelography (IVP).^[8–10]^ Typical pictures of MSK show collections of contrast media in dilated collecting tubules with the appearance of a papillary brush or bouquet of flowers.^[8,9]^ These radiographic findings are generally used in the diagnosis of MSK along with medullary nephrolithiasis, medullary nephrocalcinosis, and medullary cysts.^[9]^ Although there have been several cases reporting that MSK may be involved in hypertension, whether it is an etiological factor for secondary hypertension remains unknown.

In 1977, a case of hypertension with MSK was reported in an adolescent; however, the patient was diagnosed with essential hypertension and normal renin activities.^[11]^ In 2008, another case of MSK combined with hypertension was presented by Michele.^[12]^ This patient was found to have left kidney atrophy and was then diagnosed with renovascular hypertension for the stenosis of the left renal artery. A pathological biopsy after the left nephrectomy confirmed the presence of MSK and renal artery fibromuscular dysplasia (FMD). It was considered that FMD - but not MSK - was an etiological factor for hypertension. Our patient with susceptive secondary hypertension exhibited secondary aldosteronism combined with MSK. The common causes related to secondary aldosteronism, such as renal arteries stenosis, glomerulonephritis, diabetic nephropathy and lupus nephropathy, have been excluded. Therefore, the etiology of hypertension was thought to be related to MSK. The pathophysiologic mechanism involved in may be the over-activation of renin-angiotensin-aldosterone system (RAAS), since the perfusion of the right kidney was reduced slightly, as confirmed via renal dynamic imaging. Hypoperfusion was supposed to be the consequence of the compression of the renal arterioles caused by the dilation of the collecting ducts and hydronephrosis.

In addition, the possibilities of PCOS and drug-related hypertension were also suspected. PCOS is a common endocrinopathy in women of reproductive age.^[13]^ It has been suggested that the diagnosis of PCOS should be made if two of the following three criteria are met: androgen excess, ovulatory dysfunction, polycystic ovaries.^[14]^ The relationship between PCOS and hypertension has remained controversial until now. Retrospective studies have shown that patients with PCOS have a higher incidence of hypertension and their BP is significantly higher than that of control groups.^[15–17]^ However, a prospective study with a 12-year follow-up period published by Tehrani showed that the BP of PCOS patients was slightly higher than in control groups, yet there was no statistically significant difference.^[18]^ For our patient, PCOS was excluded because there was neither clinical or biochemical hyperandrogenism nor polycystic ovaries excepted for menopause-like symptoms. Besides, Dine-35 as an oral contraceptive may result in hypertension due to the effect of estrogen, which could lead to sodium and water retention or activate angiotensinogen. A meta-analysis showed a linear relationship between the risk of hypertension and the duration of an oral contraceptive treatment. The relative risk (RR) of hypertension was 1.01 in patients with 0.5 years of treatment.^[19]^ Therefore, it was considered that the risk of drug-related hypertension in our patient was lower, because she took the drug for only 21 days and had stopped for two months prior to admission.

In conclusion, this case indicated that medullary sponge kidney may be associated with secondary hypertension. The mechanism may be the activation of RAAS caused by the low perfusion of the kidney. Moreover, it was shown that angiotensin-converting enzyme inhibitors (ACEI) could effectively control the patient's BP and improve secondary aldosteronism due to the vasodilation of the afferent arterioles.

## Author contributions

**Conceptualization:** Wei Song.

**Data curation:** Ying Zhang.

**Investigation:** Fengyuan Wu, Yunpeng Cheng.

**Project administration:** Yan Lu.

**Supervision:** Yinong Jiang.

**Validation:** Yinong Jiang.

**Writing – original draft:** Fengyuan Wu.

**Writing – review & editing:** Wei Song.
